# Characterization of novel isoforms and evaluation of *SNF2L/SMARCA1 *as a candidate gene for X-linked mental retardation in 12 families linked to Xq25-26

**DOI:** 10.1186/1471-2350-9-11

**Published:** 2008-02-26

**Authors:** Maribeth A Lazzaro, Matthew AM Todd, Paul Lavigne, Dominic Vallee, Adriana De Maria, David J Picketts

**Affiliations:** 1 Ottawa Health Research Institute, 501 Smyth Road, Ottawa, ON K1H 8L6, Canada; 2 Health Canada, Therapeutic Products Directorate, Bureau of Cardiology, Allergy and Neurological Sciences, Tunney's Pasture, Ottawa, Ontario K1A 1B9, Canada; 3 University of Ottawa Centre for Neuromuscular Disease and Departments of Medicine, Biochemistry, Microbiology and Immunology, Ottawa, ON, K1H 8M5, Canada

## Abstract

**Background:**

Mutations in genes whose products modify chromatin structure have been recognized as a cause of X-linked mental retardation (XLMR). These genes encode proteins that regulate DNA methylation (*MeCP2*), modify histones (*RSK2 *and *JARID1C*), and remodel nucleosomes through ATP hydrolysis (*ATRX*). Thus, genes encoding other chromatin modifying proteins should also be considered as disease candidate genes. In this work, we have characterized the *SNF2L *gene, encoding an ATP-dependent chromatin remodeling protein of the ISWI family, and sequenced the gene in patients from 12 XLMR families linked to Xq25-26.

**Methods:**

We used an *in silico *and RT-PCR approach to fully characterize specific SNF2L isoforms. Mutation screening was performed in 12 patients from individual families with syndromic or non-syndromic XLMR. We sequenced each of the 25 exons encompassing the entire coding region, complete 5' and 3' untranslated regions, and consensus splice-sites.

**Results:**

The *SNF2L *gene spans 77 kb and is encoded by 25 exons that undergo alternate splicing to generate several distinct transcripts. Specific isoforms are generated through the alternate use of exons 1 and 13, and by the use of alternate donor splice sites within exon 24. Alternate splicing within exon 24 removes a NLS sequence and alters the subcellular distribution of the SNF2L protein. We identified 3 single nucleotide polymorphisms but no mutations in our 12 patients.

**Conclusion:**

Our results demonstrate that there are numerous splice variants of SNF2L that are expressed in multiple cell types and which alter subcellular localization and function. *SNF2L *mutations are not a cause of XLMR in our cohort of patients, although we cannot exclude the possibility that regulatory mutations might exist. Nonetheless, *SNF2L *remains a candidate for XLMR localized to Xq25-26, including the Shashi XLMR syndrome.

## Background

The isolation of genes underlying X-linked mental retardation (XLMR) disorders has been hampered, in part, by the broad phenotypic variability observed in patients that restricts linkage analysis to large single families or instances where a specific trait facilitates phenotypic splitting. More recently, the use of large scale genomic methods including comparative genome hybridization (CGH) arrays and patient sequencing projects has increased the identification rate of XLMR disease genes. Surprisingly, each gene identified accounts for a small proportion of cases and there remain many conditions for which a gene has not been identified. Nonetheless, several trends have emerged. These include the identification of XLMR genes encoding proteins that modulate chromatin structure [1]. The cloning of the *ATRX *gene as the cause of the α-thalassemia mental retardation (ATR-X) syndrome established the paradigm for chromatin remodeling proteins in neurodevelopmental disorders [2]. This gene, encoding a SWI/SNF-like protein, is also mutated in other severe XLMR syndromes lacking α-thalassemia and in patients with mild-to-moderate XLMR [3]. Subsequently, the *RSK2 *gene encoding a histone kinase was identified as the causative gene for Coffin-Lowry syndrome and non-specific XLMR [4,5], and the methyl-CpG-binding protein 2 (*MeCP2*) gene was identified as the causative gene for Rett syndrome [6] and other non-specific male MR [7-9]. More recently, the *PHF6 *(Borjeson-Forssman-Lehmann syndrome;[10]), *ZNF41 *[11], *ZNF81 *[12], and *JARID1C *[13] genes have also been implicated in XLMR and have roles in transcriptional regulation and/or chromatin remodeling. Taken together, these studies suggest that additional chromatin interacting proteins whose genes reside on the X chromosome should be considered as disease candidates for both syndromal and non-specific XLMR disorders.

The Drosophila ISWI gene was identified as a distinct SWI/SNF subclass named the Imitation SWI (ISWI) family [14]. Two human orthologs of *Drosophila *ISWI (dISWI) have been described, *SNF2H *(SMARCA5) which maps to 4q31.1 and *SNF2L *(SMARCA1) which maps to Xq25-26 [15,16]. Moreover, analysis of the murine *Snf2h *and *Snf2l *genes demonstrated that *Snf2h *was expressed in proliferating neuroblast layers whereas *Snf2l *expression was enhanced in differentiating neuronal populations [17]. Indeed, purification of the SNF2L-containing human NURF complex demonstrated that it regulated expression of the *engrailed *genes, which are important in mid/hind-brain development [18]. In addition, the latter study also demonstrated that SNF2L could promote neuronal differentiation when expressed ectopically in neuroblasts [18]. SNF2L was also found to be a component of a second chromatin remodeling complex, called CERF that contains the CECR2 protein, a transcription factor involved in neurulation and a cause of exencephaly in mice when mutated [19]. These studies suggest that *SNF2L *is an excellent candidate gene for the cause of XLMR. In this study, we have characterized multiple splice forms and examined 12 families with XLMR for mutations in *SNF2L*.

## Methods

### Reverse Transcription-PCR

For cell lines, total RNA was prepared from cell lines by acid phenol extraction of cell lysates [20]. Poly A+ RNA for reverse transcription was purified from total RNA using the PolyATtract mRNA Isolation System (Promega, Nepean, Ontario). Total RNA from human tissues and specific brain regions were obtained commercially (Applied Biosystems Canada, Streetsville, Ontario). Total RNA (2 ug) or Poly A+ RNA (100 ng) was reverse transcribed using Superscript RT (Invitrogen) and a combination of oligo dT and random hexamers. PCR reactions for analysis of *SNF2L *splice variants were at 94°C for 30 seconds, 53°C for 30 seconds and 72°C for 2 minutes for 35 cycles, followed by a final extension of 15 minutes at 72°C. For the 5' splice variants, the following primers were used: 5'UTR SNF2L1 Fwd, 5' CAAACTTGCTGCTAAAGCGCC 3'; 5'UTR SNF2L2 Fwd, 5' GGAATTCATGGAGCAGGACACTGC 3'; SNF2L5'splice variants Rev, 5'CACCAAGACAATTTTTAGTG 3'. For the NLS splice variants: SNF2L NLS Fwd, 5' GGAGGTCATGGAGTATTC 3' and SNF2L NLS Rev, 5'CAGTAGCTGACTCTGCTTTTCTTTTCTGTG 3'.

### Patient Material

DNA samples from 12 individuals affected with XLMR and previously mapped to a region encompassing the *SNF2L *gene were used for direct sequencing analysis. Samples from families F85-19 [21], F93-04, and F91-02 were provided by Drs. Ben Hamel and Hans van Bokhoven (University Hospital Nijmegen, Nijmegen, Netherlands). Samples K8045 [22], K8135 [23], K8320, K8395, K8725, K8895 [24], and K8923 were generously provided by Dr. Charles Schwartz (JC Self Research Institute, Greenwood Genetic Center, Greenwood South Carolina). The non-syndromic XLMR sample 24981 was provided by Dr. Judith Allanson (Children's Hospital of Eastern Ontario, Ottawa Ontario). The Pettigrew syndrome DNA sample was prepared from a commercially available EBV transformed lymphoblast cell line (GM12523; Coriell Cell Repository, Camden, NJ).

### Plasmid constructs

The SNF2L NLS splice variants were cloned into the pcDNA3 expression plasmid (Invitrogen Canada Inc. Burlington, ON), each with an amino terminal tag encoding the FLAG epitope. Annealed primers encoding the FLAG epitope were inserted in the *Kpn*I and *Eco*RI sites of the pcDNA3 plasmid. The 5' region encoding the amino terminus of the SNF2L2 splice variant (SNF2LB) was engineered with an *Eco*RI restriction site for cloning in frame with the FLAG epitope. The primers used were: FLAG-sense EcoRI, 5'-CCCACCATGGATTACAAGGATGACGACGATAAGG-3' and FLAG-antisense EcoRI, 5'-AATTCCTTATCGTCGTCATCCTTGTAATCCATGGTGGGGTAC-3'. The remainder of each cDNA (+ NLS or -NLS) was inserted in two additional cloning steps to construct plasmids encoding full length SNF2L proteins with and without a nuclear localization signal.

### Cell transfections and immunofluorescence

293HEK cells were cultured in EMEM (Eagle's Minimal Essential Medium, Wisent, St Bruno, Quebec) supplemented with 10% fetal bovine serum (FBS) in a humidified 95% air with 5% CO_2 _incubator at 37°C. One day prior to transfection, cells were plated on glass coverslips coated with poly D-lysine (100 μg/ml, Sigma, Oakville, Ontario) in 6 well tissue culture plates at a density of approximately 3 × 10^5 ^cells/well. Cells were transfected with 5 μg DNA [pcDNA3 control, pcDNA3 FLAG-SNF2LΔNLS, or pcDNA3 FLAG SNF2L] and the Lipofectamine 2000 reagent (Life Technologies, Burlington, Ontario) according to the manufacturer's protocol. After 48 hours, cells were fixed in cold ethanol/methanol (3:1) prior to blocking with 10% FBS diluted in PBS. Coverslips were incubated with diluted primary antibody (murine anti-FLAG monoclonal clone M2, 2 μg/ml, Sigma, Oakville, Ontario) for 1 hour at room temperature, washed and then incubated for 1 hour at room temperature with FITC-conjugated donkey-anti-murine IgG secondary antibody (Sigma) diluted 1:100 in PBS. Cell nuclei were counterstained with 1 μg/ml DAPI (Sigma) and images were captured using a Zeiss Axioplan 2 microcope outfitted with an AxioCam camera and AxioVision software.

### SNF2L Mutation Analysis

Genomic DNA from individuals affected with XLMR in each of the 12 families analyzed was used to amplify each exon of the *SNF2L *gene with forward and reverse primers that annealed to flanking intron sequences, approximately 100 bp from each exon/intron boundary (primer sequences available upon request). Genomic DNA from an individual unaffected by XLMR was the control for all PCR reactions. For each reaction, 100–200 ng DNA was combined with 1 μM each of forward and reverse PCR primers, 200 μM dNTPs, 2.5 mM MgCl_2_, 20 mM Tris-HCl pH 8.4, 50 mM KCl, and 1 unit Taq DNA polymerase in a volume of 100 μl. PCR reactions were incubated at 95°C for 40 seconds, 57°C for 40 seconds and 72°C for 3 minutes for 30 cycles, followed by a final extension of 15 minutes at 72°C. DNA obtained from PCR reactions was purified using the Qiaquick PCR Purification kit (Qiagen, Mississauga, Onatario) and sequenced using Perkin Elmer dye terminator and ABI automated sequencing. Sequences were aligned and compared to sequences obtained from the control DNA and sequence available in public databases to identify mutations.

## Results and Discussion

**Figure 1:**
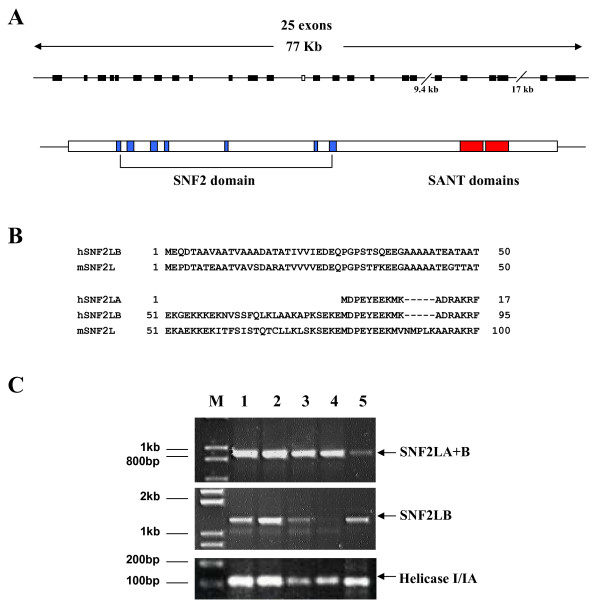
**Genomic organization and 5' transcript variants of the human *SNF2L *gene**. **A**. Schematic diagram showing the 25 exons (dark boxes; exon 13 is an open box) of the human *SNF2L *gene (top). Below is a schematic diagram of the SNF2L transcript showing the ORF (open box) and the location of the motifs that comprise the SNF2 domain (blue boxes) and the SANT domains (red boxes). **B**. The 5' variants *SNF2LA *and *SNF2L*B provide alternative initiation codons and encode two forms of *SNF2L *with different amino-termini. They are shown aligned to the mouse *Snf2l *sequence. The *SNF2L*B transcript encodes a protein with an amino-terminus similar in length and amino acid composition to the murine Snf2l protein. **C**. RT-PCR analysis showing that both transcript variants are present in human cell lines and fetal brain tissue examined. The helicase I/Ia domain served as control amplification. Lane 1, 293 cells; lane 2, SH-SY5Y cells; lane 3, NT2 cells; lane 4, hNT neurons; and lane 5, human fetal brain. M, molecular weight marker.

Spatial and temporal expression studies of the *Snf2l *gene in mice and the purification of two human SNF2L-containing complexes have both suggested that the SNF2L protein may have an important role in neurodevelopment and that the *SNF2L *gene is a strong XLMR candidate gene [17-19]. *In silico *analysis demonstrated that the *SNF2L *gene (NM_003069) is highly conserved between mouse and human with a common intron/exon pattern containing 25 exons spanning ~77 kb along the X chromosome within Xq25 (Figure 1A and data not shown). However, the human cDNA and predicted protein sequences showed several significant discrepancies to the mouse *Snf2l *sequence (NM_053123) that required characterization, prior to mutation studies [16,17]. Indeed, we previously reported the presence of a human *SNF2L *variant (SNF2L+13) containing a non-conserved in-frame exon within the SNF2 catalytic domain that abolishes chromatin remodeling activity [25]. In addition, Okabe et al. reported two human cDNA clones with disparate 5' ends [16]. We will refer to these two clones as *SNF2LA *and *SNF2LB*, respectively. *SNF2LB *aligns with the start of the murine cDNA sequence and corresponds to a transcript that would initiate within exon 1 of the human genomic sequence (Figure 1B). The shorter *SNF2LA *isoform results from transcription that initiates within exon 2. The corresponding proteins differ in size at the NH_2_-terminus by 78 amino acids with *SNF2LB *corresponding to the published murine sequence (Figure 1B). The *SNF2L*A isoform was not present in mice suggesting that it may be unique to humans. Using RT-PCR, we detected the corresponding transcripts for both 5' variants in human fetal brain and multiple human neuronal cell lines (Figure 1C).

**Figure 2:**
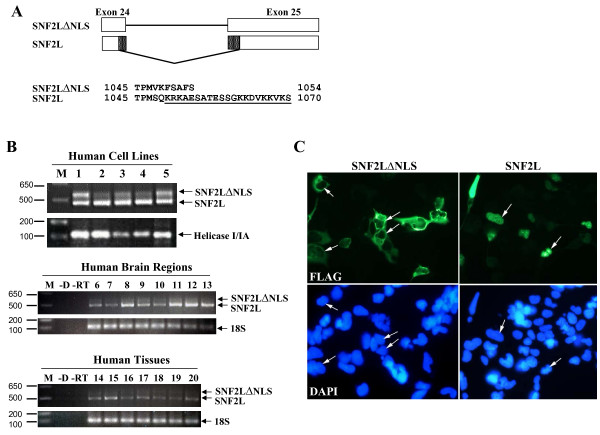
**Alternative splicing of exons 24 and 25 generates nuclear and cytoplasmic isoforms of SNF2L**. Alternative splicing of the *SNF2L *gene at the 3' end generates a transcript containing either the full sequence of exons 24 and 25, which encodes a shorter form of SNF2L without a nuclear localization signal (SNF2LΔNLS), or a transcript lacking 100 bp that encodes for a larger protein isoform (SNF2L) with an NLS (underlined). **B**. RT-PCR analysis demonstrated that both 3' variants are present in most cells and tissues examined, while the NLS isoform is predominant. M, marker; -D, no DNA template; -RT, no reverse transcriptase. Lanes 1–5, human cell lines and fetal brain sample as follows: 1, 293-HEK cells; 2, SH-SY5Y cells; 3, NT2 cells; 4, hNT neurons; 5, human fetal brain. Lanes 6–13, human brain regions including: 6, amygdala; 7, basal ganglia; 8, caudate nucleus; 9, cerebellum; 10, frontal cortex; 11, hippocampus; 12, pons; 13, thalamus. Lanes 14–20, human tissue samples including: 14, heart; 15, kidney; 16, liver; 17, ovary; 18, placenta; 19, skeletal muscle; and 20, testes. **C**. Indirect immunofluorescence imaging of 293 HEK cells transfected with FLAG-epitope tagged SNF2LΔNLS and SNF2L were stained with anti-flag antibody (green) or DAPI (blue). Note that SNF2LΔNLS encodes a protein that is localized exclusively in the cytoplasm while SNF2L is expressed only in the nucleus (arrows point to nuclei of transfected cells).

In addition to the variation at the amino-terminus, the human and mouse amino acid sequences also differed at their carboxyl-terminus following Blast tool analysis. The last seven amino acids of the human sequence were not present in the mouse SNF2L protein sequence, but instead were replaced by 23 unique residues (Figure 2A). RT-PCR analysis demonstrated that both 3'-end variants could be detected in a variety of human cell lines, tissues and specific brain regions with the NLS-containing isoform representing the predominant species (Figure 2B). Sequencing demonstrated that the two transcripts are generated by alternative splicing of exons 24 and 25. Inclusion of exons 24 and 25 result in a shorter peptide due to a stop codon located at the start of exon 25. We called this variant SNF2LΔNLS. Alternative use of splice sites within exons 24 and 25 removes 100 bp and generates a transcript that contains an additional 23 amino acids similar to the reported mouse protein (and herein called SNF2L). More importantly, we used the PredictNLS program [26] to determine that this alternatively spliced region contained a putative NLS sequence. To verify the importance of the NLS *in vivo*, we transiently transfected 293 HEK cells with FLAG-epitope tagged SNF2L or SNF2LΔNLS and detected the expressed protein by indirect immunofluorescence. As shown in Figure 2C, SNF2LΔNLS localized exclusively in the cytoplasm whereas SNF2L was present in the nucleus.

The *SNF2L *gene undergoes alternative splicing at multiple sites to generate a wide number of different isoforms. Some of these isoforms will affect the activity of the protein. For example, the inclusion of exon 13 abolishes the chromatin remodeling activity [25], and the altered splicing within exons 24/25 removes the NLS resulting in cytoplasmic localization. In the latter case, the SNF2LΔNLS variant was detected in most tissues examined, suggesting that the cytoplasmic isoform of SNF2L may fulfill an alternative role to the chromatin remodeling function of the nuclear isoforms. Alternatively, it could represent a SNF2L dominant negative protein that sequesters other components of the chromatin remodeling complex in the cytoplasm. As such, it will be important to further explore the function of these novel SNF2L isoforms to determine their specific roles, similar to work done with the SNF2L+13 variant. Indeed, with splicing occurring at the 5'-end of the gene (isoforms SNF2LA and B), the 3'-end of the gene (NLS or ΔNLS) and encompassing exon 13 (+exon 13 or -exon 13) there are 8 possible isoforms of the SNF2L protein that can impinge on the function/activity of the protein.

We have demonstrated that expression of the murine *Snf2l *gene increases during development in a manner that is coincident with neuronal maturation and synapse formation [17]. This expression profile is most prevalent in the hippocampus, dentate gyrus and the cerebellum and expression within these structures is maintained in the adult mouse [17]. Given that the hippocampus and dentate gyrus have been implicated in learning and memory, and that mutations in genes involved in chromatin remodeling or modification cause mental retardation, it prompted us to examine the human *SNF2L *gene as a candidate XLMR gene. DNA samples from 12 individuals affected with XLMR and previously mapped to a region encompassing the *SNF2L *gene were used for direct sequencing analysis. We sequenced each of the 25 exons and flanking intron sequences of the *SNF2L *gene and identified 3 different single nucleotide polymorphisms (SNPs), all located in untranslated sequences (Table 1). Screening of 100 unaffected individuals demonstrated that the SNP in the 5' UTR of exon 1 and the polymorphism in intron 13 (rs2274093) had frequencies of 13% and 15% respectively, in our control population. Conversely, the SNP in intron 18 was not found in our control DNA samples, but screening of the SNP database identified it as a polymorphism with a heterozygosity frequency of 0.093 (rs3736692). Overall, the SNP database contains 195 entries for the *SNF2L *gene including 5 in the coding region (exons 1, 10, 16, 17, and 20). The SNP within exon 20 is reported to have a heterozygosity value of 0.026 and would result in an Ala to Gly substitution. While we did not find this change in any of our families, future analysis of this gene should be wary of this polymorphism.

**Table 1:** Mutation analysis of 12 families linked to Xq25 revealed 3 SNPs

**Location of SNP**	**SNP**	**Family**	**% of control sample with SNP (n = 100)**	**SNP Heterozygosity (SNP Database No.)**
Exon 1 – 5'UTR 29 bp 5' of AUG	CTTGTCCC CTTATCCC	F93-04	13%	ND
Intron 13 48 bp 5' of exon14	CAACAGTA CAATAGTA	K8895	15%	0.359 (rs2274093)
Intron 18 74 bp 3' of exon18	CAGATTTAC CAGATTTTC	24981	0%	0.093 (rs3736692)

Despite the absence of mutations in these families, SNF2L remains a logical candidate for XLMR localized to Xq25-26 because of (a) its similarity to *ATRX*, a SNF2-domain containing protein mutated in several XLMR disorders; (b) its high expression in the brain including areas important for learning and development; and (c) its ability to induce neuroblastoma cells to undergo differentiation when ectopically expressed [17,18]. In addition, SNF2L has been shown to regulate the engrailed genes, En-1 and En-2, both of which are important for mid/hind-brain development [27,28], the latter of which has been associated with autism in genetic linkage studies [29-31]. Given these criteria and the small sample size available for mutation analysis, other syndromes should be considered for screening, including the Shashi XLMR syndrome [32,33] and a family with syndromal XLMR and late-onset testicular failure (Cillier syndrome) [34] that both map to Xq26. In addition, there are 3 other syndromes (Wilson/MRXS12, Gustavson, and CMTX4/Cowchock-Fishbeck) and 8 MRX families (MRX 27, 35, 42, 62, 70, 71, 75, and 82) that map to this region that should also be considered in future screening endeavors [35]. The generation of a transgenic mouse ablated for the *Snf2l *gene should provide valuable phenotypic insight for its potential involvement in specific XLMR disorders. Together, the analysis of additional samples and the characterization of transgenic mice should define whether the SNF2L gene is a cause of mental retardation.

## Conclusion

We have shown that there are multiple SNF2L isoforms that result from alternative splicing of the gene. How these different isoforms are spatially and temporally regulated and defining their specific role during neural development remains to be established. In our collection of 12 patients with XLMR linked to Xq25-26, we did not identify any mutations within the coding region. However, we cannot exclude intronic mutations (outside of the consensus splice sites) that would affect alternative splicing and hence the expression levels of the different isoforms. Similarly, we cannot exclude mutations in regulatory regions in the 12 families screened for mutations in this gene. Alternatively, our failure to identify mutations may arise from (1) the small sample size which may have prevented us from ascertaining a family with a mutation in this gene, or (2) the possibility that mutations in SNF2L could cause a more severe phenotype that may be lethal in males. Indeed, SNF2L remains a candidate XLMR gene for Xq25-26 linked XLMR families including the Shashi syndrome as well as in sporadic mental retardation cases.

## Competing interests

The author(s) declare that they have no competing interests.

## Authors' contributions

MAL identified the various SNF2L isoforms, generated the preliminary RT-PCR data and performed the immunofluorescence experiments. MAMT extended the RT-PCR experiments to include the multiple human tissues and brain regions. PL and DV performed the sequence analysis of the XLMR patients. AD prepared the initial figures and wrote the first draft of the manuscript. DJP designed the study, participated in its execution and coordination and wrote the final manuscript. All authors have read and approved the final manuscript.

## Pre-publication history

The pre-publication history for this paper can be accessed here:


